# Microglia: Agents of the CNS Pro-Inflammatory Response

**DOI:** 10.3390/cells9071717

**Published:** 2020-07-17

**Authors:** José A. Rodríguez-Gómez, Edel Kavanagh, Pinelopi Engskog-Vlachos, Mikael K.R. Engskog, Antonio J. Herrera, Ana M. Espinosa-Oliva, Bertrand Joseph, Nabil Hajji, José L. Venero, Miguel A. Burguillos

**Affiliations:** 1 Institute of Biomedicine of Seville (IBIS)-Hospital Universitario Virgen del Rocío/CSIC/University of Seville, 41012 Seville, Spain; rodriguez@us.es (J.A.R.-G.); ajherrera@us.es (A.J.H.); anaespinosa@us.es (A.M.E.-O.); jlvenero@us.es (J.L.V.); 2 Department of Medical Physiology and Biophysics, Faculty of Medicine, University of Seville, 41009 Sevilla, Spain; 3 Department of Biochemistry and Molecular Biology, Faculty of Pharmacy, University of Seville, 41012 Seville, Spain; ekavanagh@us.es; 4 Institute of Environmental Medicine, Toxicology Unit, Karolinska Institute, 17177 Stockholm, Sweden; pinelopi.vlachos@ki.se (P.E.-V.); bertrand.joseph@us.es (B.J.); 5 Department of Medicinal Chemistry, Analytical Pharmaceutical Chemistry, Uppsala University, 751 23 Uppsala, Sweden; mikael.engskog@ilk.uu.se; 6 Division of Brain Sciences, The John Fulcher Molecular Neuro-Oncology Laboratory, Imperial College London, London W12 ONN, UK; n.hajji@imperial.ac.uk

**Keywords:** microglia, inflammation, TLR4, TREM2, caspases, epigenetics, metabolomics, iPSCs

## Abstract

The pro-inflammatory immune response driven by microglia is a key contributor to the pathogenesis of several neurodegenerative diseases. Though the research of microglia spans over a century, the last two decades have increased our understanding exponentially. Here, we discuss the phenotypic transformation from homeostatic microglia towards reactive microglia, initiated by specific ligand binding to pattern recognition receptors including toll-like receptor-4 (TLR4) or triggering receptors expressed on myeloid cells-2 (TREM2), as well as pro-inflammatory signaling pathways triggered such as the caspase-mediated immune response. Additionally, new research disciplines such as epigenetics and immunometabolism have provided us with a more holistic view of how changes in DNA methylation, microRNAs, and the metabolome may influence the pro-inflammatory response. This review aimed to discuss our current knowledge of pro-inflammatory microglia from different angles, including recent research highlights such as the role of exosomes in spreading neuroinflammation and emerging techniques in microglia research including positron emission tomography (PET) scanning and the use of human microglia generated from induced pluripotent stem cells (iPSCs). Finally, we also discuss current thoughts on the impact of pro-inflammatory microglia in neurodegenerative diseases.

## 1. Introduction

Microglia were originally termed the ‘third element of nerve centers’ by Santiago Ramón y Cajal at the beginning of the last century [1], but it was not until a few years later that his student, Pio Del Rio-Hortega, gave them their current name [2]. Microglia were originally described as macrophages resident in the central nervous system (CNS) [3,4,5,6] and, though microglia share features with peripheral macrophages, several studies have demonstrated the difference in ontology between bone marrow-derived macrophages and microglia. Microglia progenitor cells originate in the yolk sac [7,8,9] from where they proliferate and migrate towards the CNS [10], maintaining homeostasis of the nervous tissue through permanent surveillance of the brain parenchyma [11]. Throughout this process, microglia appear to undergo three stages of development defined in Matcovitch-Natan and colleagues [12], starting from early microglia until E14, followed by microglia from E14 to a few weeks after birth, and finally adult microglia from a few weeks after birth onward. The differences in ontology between bone marrow-derived macrophages and microglia may explain some of the observed cell-type variances in the inflammatory response [13].

Since their discovery, just over a century ago [14], several roles have emerged for microglia. For instance, microglia play a key role during development, affecting the structure of several neuronal circuits such as in cerebellum [15], promoting learning-dependent synapse formation in the motor cortex [16], or removing excess of newborn cells from the neurogenic cascade of the adult hippocampus [17]. But these multi-tasking cells have also a prominent role in the etiology and progression in different neurodegenerative diseases. To illustrate this, the seminal work performed by McGeer and colleagues showed the presence of reactive microglia, based on the expression of Human Leukocyte Antigen—**DR** isotype (HLA-DR), and other immune cells like T cells, in the brains of Alzheimer disease (AD) and Parkinson disease (PD) patients [18,19,20]. These studies laid the foundations for considering neuroinflammation a potential factor involved in the onset and/or development of these disorders. After those studies, multiple markers apart from HLA-DR, whose expression is high in microglia in multiple sclerosis patients [21], have been used to describe markers of reactive microglia such as CD68 [22] and CD105/Endogolin, a co-receptor for transforming growth factor beta (TGFβ) receptor that antagonizes TGFβ signaling and is found in microglia associated with degenerating dopaminergic neurons and neuromelanin [23].

In this review we will discuss different aspects of microglial pro-inflammatory response. It is difficult to accurately define the “pro-inflammatory microglia” phenotype as various stimuli and brain conditions elicit similar yet distinct microglia responses, as recently shown by Friedman and colleagues [24]. However “pro-inflammatory microglia” can be considered to be highly pro-oxidant [25]. In this context we understand the “pro-inflammatory response” as the immune response whose over-activation may cause neurotoxicity. In principle, the pro-inflammatory response is not a deleterious response but a necessary one under physiological conditions. An acute inflammatory process driven by microglia begins as a “defense and repair” mechanism when a challenge disrupts brain homeostasis. This acute inflammatory response works under tight control mechanisms that ensure microglia deactivation once their task has been completed. For reasons not yet fully understood, acute inflammation sometimes switches into an endlessly self-sustaining toxic process that damages tissue instead of repairing it. For instance, in the case of neurodegenerative pathologies, the abnormal accumulation of damaged/misfolded proteins, oxidative stress, or peripheral inflammatory processes have been identified as factors contributing to the shift from acute to chronic inflammation [26].

Over time, our understanding of how microglia become active has evolved. Until recently, microglial activation was mostly understood as resting microglial cells that transformed into fully activated cells [27,28] through two possible activation mechanisms: A classic M1 state that produced pro-inflammatory cells, following the terminology used in activating peripheral macrophages and Th cells, and an alternative M2 state that produced cells with an anti-inflammatory or tumor-supportive profile [29,30]. However, in light of results obtained in recent genetic data analyses [31,32], this method of classification required revision of the restrictive M1/M2 activation paradigm to adequately describe the rich diversity of microglial response. According to this view, the numerous functions of microglia would be fulfilled through acquisition of multiple phenotypes, each associated with a distinct molecular signature [33,34,35,36,37,38,39,40,41,42,43]. Recent evidence also suggests that different pools of microglia might each display distinct intrinsic properties that would be acquired during their maturation or function within the CNS. Therefore, it has been recently proposed that microglia might constitute a community of cells in which different populations display distinct properties, perform distinct physiological functions, and respond differently to stimuli [44].

We aimed in this review to highlight key aspects of the pro-inflammatory response in microglia with a focus on their contribution to neurodegenerative disease. We will begin by introducing the reader to pro-inflammatory signaling cascades in microglia, describing key players in the pro-inflammatory response during neurodegeneration as well as commenting on recent discoveries on the contribution of microglia to neuroinflammation. This will be followed by a discussion of epigenetic regulation of inflammation, immunometabolism, and new technologies to overcome the current challenges of microglia research.

## 2. Microglia Pro-Inflammatory Signaling

As the primary immune cell type of the CNS, microglia are constantly surveying their environment for deleterious signals of foreign (bacterial or viral) or endogenous (DNA, RNA, protein) origin. These stimuli are recognized by pattern recognition receptors (PRRs) located, with some exceptions, on the plasma membrane of microglia. PRRs include toll-like receptors (TLR), inflammasome-forming nucleotide-binding oligomerization domain (nod)-like receptors (NLRs), and triggering receptor expressed on myeloid cells (TREMs), among others. This ligand–receptor interaction triggers a series of signaling events resulting in the production of inflammatory factors including interleukin-1 β (IL-1β), IL-6, tumor necrosis factor-α (TNFα), cyclooxygenase-2 (COX2), nitric oxide synthase 2 (NOS2), reactive oxygen species (ROS), and the release of acute-phase proteins such as pentraxin-3 [45], a protein whose expression is triggered by several TLRs’ ligands [46] and facilitates microglial phagocytic activity [45,47]. Additionally, the acute inflammatory response in microglia promotes phagocytosis of nearby damaged neurons and neurotoxic aggregates in vitro and in vivo [48,49]. Microglial phagocytosis of amyloid-β and α-synuclein in neurodegenerative conditions results in their engulfment and clearance mediated by receptors such as β1 integrin or TLR4 [50,51]. This points to a protective role of microglia inflammatory signaling in neurodegeneration. However, although LPS and neurotoxic aggregates initially promote microglial phagocytosis [52,53], chronic activation of microglia impairs their phagocytic capacity, resulting in reduced aggregate clearance during neurodegeneration [54,55,56]. Conversely, though impaired microglia phagocytosis may contribute to neurotoxic aggregate buildup, inappropriate phagocytosis of neurons occurs during neurodegeneration via complement system opsonization of neuronal synapses [57,58].

This acute inflammatory response is a necessary defense mechanism against nearby infected, diseased, or damaged cells. However, if this response becomes prolonged or chronic, it may exacerbate neurodegeneration. Activated microglia are a pathological feature of neurodegenerative diseases such as AD, PD, and amyotrophic lateral sclerosis (ALS), and targeting these aspects of microglia activation are of therapeutic interest [59]. In this section, we will review microglia pro-inflammatory signaling pathways including those with relevance to neurodegenerative disease.

### 2.1. TLR4 Pro-Inflammatory Signaling

Immune detection of deleterious stimuli, whether they be of foreign origin or not, is carried out by PRRs, each with specific affinities for pathogen associated molecular patterns (PAMPs) such as bacterial, fungal, or viral components or damage-associated molecular patterns (DAMPs), such as endogenous nucleic acid material and proteins [60]. Within the TLR family of PRRs, several are expressed by microglia cells (see Table 1) and drive the neuroinflammatory response. Although a number of TLRs are linked to neuroinflammatory processes in neurodegenerative diseases, for the purposes of this review we focused on TLR4, as this is the best characterized in terms of signaling and pro-inflammatory response in microglia (for a more comprehensive review on TLRs see [61,62]). Of the many PAMPs and DAMPS that can activate TLR4 (see Table 1), some have particular relevance to neurodegenerative disease such as amyloid-β (Aβ) [63], α-synuclein [51] and galectin-3 (described in further detail below). Indeed, mouse studies have shown that toxic, aggregated conformations of amyloid-β and α-synuclein can promote microglia activation via activation of TLR4 (amyloid-β) or TLR2 (amyloid-β and α-synuclein) [63,64]. TLR4 expression itself is increased in patients with AD [65] as well as being increased in microglia surrounding plaques. Inhibition of TLR4 activation and signaling is beneficial in several animal models of neurodegenerative disease (for review see [66]).

**Figure 1:**
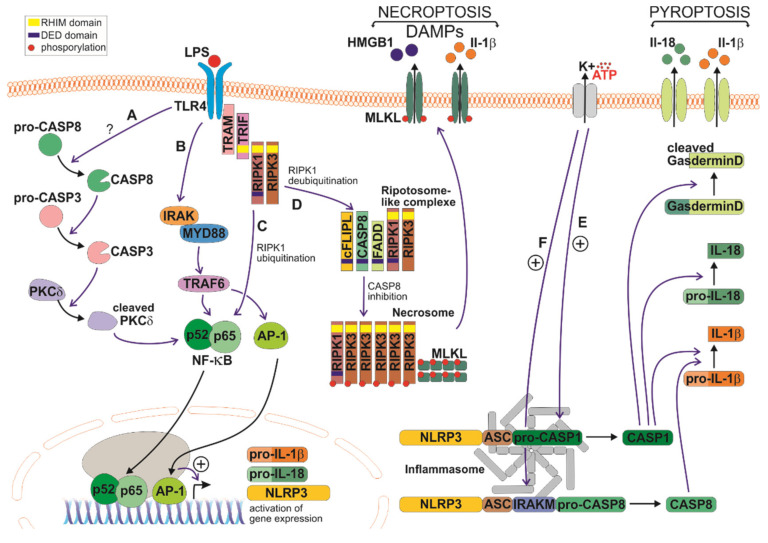
TLR4 signaling pathways activated in microglia during neuroinflammation. (**A**) LPS binding to TLR4 triggers sequential activation of caspase-8 and caspase-3 with nuclear translocation of NF-κB and expression of genes involved in inflammatory response. The molecular mechanism triggering activation of caspase-8 is unknown at the moment. LPS can also activate the expression of inflammatory genes by means of (**B**) the MyD88-dependent pathway or (**C**) the TIR-domain containing adapter-inducing interferon-γ (TRIF)-dependent pathway with receptor-interacting protein kinase 1 (RIPK1) ubiquitination. (**D**) Although not clearly defined in microglia, under deubiquitinating conditions, RIPK1 can form a ripoptosome-like complex that ultimately leads to necrosome formation and necroptotic cell death with release of DAMPs. (**E**) TLR4-mediated increase in gene expression of NOD-, LRR- and pyrin domain-containing protein (NLRP3), pro-IL-1β, and pro-IL-18 is the priming stage of inflammasome formation. In the activation stage, the assembly of inflammasome complex activates caspase-1, which allows the maturation of IL-1β and IL-18 and their release through pyroptosis. (**F**) A noncanonical inflammasome has been also described in microglia that gives rise to caspase-8 activation and IL-1β maturation and release.

TLR4 activation and signaling is very complex and has been extensively covered by others [61,62]. Much of our understanding of signaling cascades comes from the use of the bacterial cell wall component lipopolysaccharide (LPS) as a TLR4 activator [96] (see Figure 1). LPS binding to TLR4 and its co-receptor CD14 leads to receptor dimerization and recruitment of adaptor proteins to the plasma membrane, resulting in two possible signaling cascades. In the first, myeloid differentiation primary response 88 (MyD88) interacts with the active TLR4 homodimer complex through its toll-interleukin receptor (TIR) domain and recruits interleukin-1 receptor-associated kinase (IRAK) and the really interesting new gene (RING)-domain E3 ubiquitin ligase, TNF receptor-associated factor (TRAF6). Ubiquitination of TRAF6 itself and the TAK1 protein kinase complex results in TAK1 activation. TAK1 then activates two different pathways that lead to activation of the inhibitor of nuclear factor kappa B kinase (IKK) complex nuclear factor kappa light chain enhancer of activated B cells’ (NF-κB) pathway and mitogen-activated protein kinase (MAPK) pathway. The IKK complex phosphorylates the NF-κB inhibitory protein, nuclear factor of kappa light polypeptide gene enhancer in B cells’ inhibitor alpha (IkBα), which undergoes proteasome degradation, allowing the translocation of the p50/p65 NF-κB heterodimer to the nucleus NF-κB to induce pro-inflammatory gene expression. NF-κB has up to 500 transcriptional gene targets, many directly related to the pro-inflammatory response including *NOS2* and mitochondrially encoded cytochrome c oxidase II (*COX2*), cytokines (including *IL-1*, *IL-2*, *IL-6*, *IL-8*, *IL-12*, *TNF*) and chemokines (*CXCL1*, *CXCL10*, *RANTES*, *MCP1*, *IL18*) [59,97]. Of the many genes transcribed, IL-1β and TNFα play an important role in pathological inflammation and the acceleration of disease [98], acting through receptors in target cells, IL1 receptor 1 (IL-1R1), and TNF receptor 1 (TNFR1), respectively [59]. TAK1 activation also results in activation of MAPK family members such as ERK1/2, p38 and JUN-amino-terminal kinase (JNK), which mediates activation of AP-1 family transcription factors [96,99,100,101]. The pro-inflammatory genes regulated by AP-1 transcription factors include *COX2* and *NOS2* [59].

Independently of MyD88, TRIF can interact with TLR4 through translocation associated membrane protein (TRAM). Through homotypic RIP homotypic interaction motif (RHIM) domain interactions, RIPK1 and RIPK3 can be recruited to form a complex with TRIF [102]. RIPK1 interacts with and activates the TAK1 complex, leading to activation of NF-κB and MAPKs and induction of inflammatory agents. Ubiquitination of RIPK1 by Pellino-1 is necessary for NF-κB activation and cytokine production in the TRIF-dependent pathway [103]. Microglia express high levels of Pellino-1, suggesting this protein as an important regulator of the neuroinflammatory response of these cells [104,105]. TRIF (in a RIPK1-independent manner) can also activate the interferon-β response through interferon regulatory factor 3 (IRF3) gene transcription [106]. 

### 2.2. TLR4 Transcriptional Targets

Although there are hundreds of genes upregulated in response to TLR4 activation, for the purposes of this review, we will highlight some that illustrate the microglia inflammatory response. We begin with TNFα, a potent pro-inflammatory cytokine [107] and a high-affinity ligand and activator of TNFα receptor (TNFR) signaling pathway, triggering a variety of downstream events including death-receptor caspase activation and apoptosis, RIPK1 kinase-mediated necroptosis, and inflammatory gene expression through NF-κB and activator protein 1 (AP-1) transcription factors [108]. Aside from the transcription of pro-inflammatory cytokines, TLR4 signaling also promotes ROS production through transcriptional upregulation of nicotinamide adenine dinucleotide phosphate (NADP)H oxidase 2 (NOX2). This is a multi-subunit enzyme complex activated in response to environmental, chemical, and infectious stimuli [109]. Located at the plasma membrane, the active NOX2 complex produces superoxide ion through a redox reaction with molecular oxygen and NADPH [110]. In activated microglial cells, the main source of ROS is NOX2 [109]. However, mitochondrial dysfunction can also contribute to ROS production by microglia [111]. ROS has not only a direct toxic impact on biological macromolecules but it also can stimulate genes which regulate the inflammatory-signaling cascades, triggering an inflammatory response [59]. Activated microglia also produce NO synthesized by NOS, with *NOS2* being a transcriptional target of TLR4 signaling through NF-κB and AP-1 [59,112,113]. Interaction between superoxide ions produced by NOX2 and NO synthesized by NOS2 gives rise to peroxynitrite causing neuronal cell death [98,114]. Therefore, the interplay between NOS2 and NOX2 seems to be key for microglia-mediated neurodegeneration. TLR4-MyD88-MAPK signaling phosphorylates and activates phospholipase A2, generating free arachidonic acid (AAc) from the plasma membrane phospholipids [115]. *COX2* is itself transcriptionally upregulated by TLR4 signaling, then catalyzes the degradation of free AAc to prostaglandin H2 (PGH2) in a two-step reaction: Firstly, dioxygenation of AAc to prostaglandin G2 (PGG2) and, secondly, peroxidation of PGG2 to PGH2. Once produced, PGH2 is transformed into prostaglandin E2 (PGE2), which is an important neuroinflammatory mediator [116]. Signal transducer and activator of transcription 3 (STAT3) is an important transcription factor in the immune system, participating in many inflammatory responses in CNS [117,118,119]. In this sense, the induction of COX2 would activate the COX2/PGE2/STAT3 pathway, thus contributing to LPS-induced IL-6 production [120].

### 2.3. TREM2 and Microglia Polarization States

As we previously stated in this review, the simplistic view that microglia polarize into two opposite polarization states (pro-inflammatory and anti-inflammatory or tumor-supportive phenotype) is outdated [32]. Within the different PRRs, there is a general consensus that TREM2 plays a central role in driving microglia activation states under disease conditions. The identification of a TREM2 rare variant, R47H, as a risk-factor gene of AD, comparable in effect to that conferred by the apolipoprotein E4 (APOE4) isoform has certainly revitalized the field, especially in the context of AD [121,122]. The further identification of a new variant (R62H) strengthened the view for a major role of TREM2 in neurodegeneration. TREM2 is a membrane receptor of the immunoglobulin superfamily whose expression within the brain is restricted to microglia [123]. TREM2 activation is mediated through interaction with protein tyrosine kinase binding protein (TYROBP, also known as DAP12). The importance of the axis TREM2/DAP12 in AD is supported by whole-genome gene expression profiling in samples obtained from patients earlier diagnosed of late-onset AD (LOAD) [124]. Molecular networks’ analysis revealed a prominent regulatory strength of the microglia module and anticipated a central role of DAP12 signaling [124]. TREM2 binds to ligands of quite different nature including an extensive range of polyanionic molecules including LPS of bacteria, glycosaminoglycans [123], phospholipids [125], APOE and apolipoprotein J (APOJ) [126,127], galectin-3 [128], apoptotic neurons [129], and Aβ [130]. Recently, a link between CD33 and TREM2 has been revealed [131]. CD33 encodes a sialic acid-binding immunoglobulin-like lectin (Siglec-3) that is exclusively expressed by microglial cells within the brain. CD33 plays important immunomodulatory roles including control of phagocytosis and suppression of cytokine release [132]. CD33, similarly to TREM2, is also considered a top risk factor for AD. Indeed, TREM2 has been reported to act downstream of CD33, affecting the expression of genes related to phagocytosis and different interleukins [131].

The advent of massive transcriptomic analysis at the single cell level allowed the identification of a novel microglia phenotype associated to neurodegenerative conditions and termed disease-associated microglia (DAM) [40]. In this study, sorting of CD45+ immune cells from brains of mice harboring 5 Familial Alzheimer Disease mutations (5xFAD) at different ages revealed three clusters of microglial cells consisting of one large cluster of homeostatic cells and two small ones associated to disease and displaying a unique molecular signature including lipid metabolism and phagocytic genes like *Apoe*, lipoprotein lipase, and cystatin F [40]. As a rule, most homeostatic genes are downregulated in DAM along with strong upregulation of select genes like *Trem2, Dap12*, *Clec7a*, *Itgax (Cd11c)*, and *Axl* [40]. The use of 5xFAD mice lacking TREM2 at different degrees of pathology identified both TREM2-dependent and TREM2-independent DAM genes. Overall, the authors concluded that the DAM program is executed in two steps, the first being TREM2-independent, followed by a TREM2-dependent program [40]. In a subsequent study, and following a similar strategy, Krasemann and colleagues isolated microglia and analyzed transcriptomes of different neurodegenerative diseases including AD (Amyloid precursor protein-Presenilin-1(APP-PS1) mice), ALS, and multiple sclerosis [41]. Overall, the study confirmed the view that homeostatic microglia express, at high levels, a subset of genes including, among others, *P2ry12*, *Tmem119*, *Hexb*, *Mertk*, and *Cx3cr1*. Under disease conditions, microglia undergo strong downregulation of homeostatic genes along with upregulation of selective genes including *Spp1*, *Itgax*, *Axl*, *Clec7a*, *Lgals3*, *Apoe* and *Grn*. This microglia polarization state was termed microglial neurodegenerative phenotype (MGnD). However, given the striking transcriptional similarities obtained in both studies, we will keep referring to this phenotype as DAM. Indeed, the existence of a common microglial disease-associated signature has been observed in several studies [133,134,135]. In AD models, DAM microglia are unequivocally associated with Aβ plaques [40,41]. However, whether this microglia phenotype plays a protective or deleterious role is a matter of intensive debate, especially since DAM microglia were described to play a neuroprotective role in [40], while, in [41], MGnD microglia (which is very similar to DAM) play a neurodegenerative role. The involvement of TREM2 in driving the DAM phenotype is inferred not only from studies in AD transgenic mice [40,136] but also in human TREM2 R47H and R62H carriers [136]. The active involvement of APOE and PGRN have been also suggested [41]. Indeed, single-cell RNAseq analysis from cortical cells obtained from individuals with different degrees of AD pathology revealed that *APOE* was strongly upregulated in microglia but downregulated in astrocytes [137]. APOE influences Aβ metabolism through modulation of the microglial immune response to amyloid-β [138], and co-localizes with microglia in a TREM2-dependent manner in APP-PS1 mice. Additionally, in AD patients with loss-of-function TREM2 variants, there is a reduction in the presence of APOE in amyloid plaques [139]. The existence of *APOE* polymorphisms have a direct impact on the risk of developing late-onset Alzheimer disease (LOAD), with *APOEε4* carriers having the greatest risk to develop AD [140]. Studies have shown higher protein expression of APOE4 in AD patients with a ε4/ε4 genotype compared to a ε3/ε4 genotype [141,142]. Based on the results in single-cell RNAseq [137] and co-localization studies with microglia [139], we inferred that APOE is a marker for microglial activation. If we assume that the TREM2/APOE axis governs the DAM phenotype, it is certainly intriguing how modulation of either TREM2 or APOE may lead to quite opposite effects. For instance, absence of TREM2 decreased amyloid pathology at early stages while increasing it at late stages [143]. Aβ plaques are toxic to surrounding neurons and TREM2 deficiency leads to increased neuritic dystrophies [144,145]. Further, TREM2 or DAP12 haplodeficient AD-like mice or AD patients with R47H mutations exhibited less compact toxic plaques thus leading to severe neuritic tau hyperphosphorylation and increased plaque-associated neuritic dystrophies [145]. Under conditions of overexpression of human TREM2 in 5xFAD mice, the number of both filamentous neurotoxic plaques and neuronal dystrophies were reduced, thus supporting the view of a protective role of TREM2 in AD pathology [146]. However, rather opposite effects have been reported in mouse models of tauopathy. Thus, TREM2 deficiency attenuated neuroinflammation and protected against brain atrophy in P301S human tau transgenic mice [147]. Interestingly, recent reports performed in P301S mice have demonstrated that overexpression of the human form of APOE4 exacerbated damage [148] and, importantly, that microglia depletion fully prevented Tau-associated damage [149]. Another DAM gene worthy of discussion is PGRN, a protein mainly secreted by microglia that modulates the inflammatory response and can influence Aβ and tau deposition [150,151]. Loss of PGRN causes frontotemporal lobar degeneration (FTLD) [150,151,152] and experimental mice lacking PGRN exhibit a quite different microglial molecular signature compared with loss of TREM2 [151]. *Trem2*^-/-^ mice provoked the increase in the expression of several homeostatic genes, whereas in the case of *Pgrn*^-/-^ microglia, there is a general repression of those same homeostatic genes. Furthermore, in APP-PS1*Pgrn*^-/-^ mice, microglia exhibited increasing phagocytic capacity, migration, and clustering around amyloid plaques. Interestingly, despite their very different transcriptomic profiles, both transgenic mice presented a reduced glucose metabolism. In both cases, “over-activation” or “over-silencing” microglia was shown to be harmful, ultimately causing neurodegeneration [151]. Overall, these studies highlight the complex roles of DAM microglia under conditions of neurodegeneration and predict the existence of different microglial subtypes ranging from neuroprotective to neurotoxic [25]. Supporting this view, the injection of systemic LPS leads to a clear pro-inflammatory molecular signature [41] different to that reported under conditions of neurodegeneration [40,41]. Comparison of transcriptomes of brain myeloid cells from various models of neurodegeneration and brain damage have identified distinct microglial modules including proliferative, interferon-related, LPS-related, and a core neurodegeneration-related [24]. Analysis of bulk AD tissue revealed the presence not only of neurodegeneration-related but also LPS-related modules [24], which is consistent with coexistence of different microglial disease-related subtypes with varying degrees of pro-inflammatory capacity [25]. 

### 2.4. Galectin-3 as a Hub Gene Associated to Neurodegeneration

The existence of multiple microglial disease-related subtypes should rely on activation of multiple PRRs. Although the involvement of TREM2 signaling in microglia polarization under disease conditions is undisputable [44,153,154], a substantial contribution of TLR signaling is expected to be relevant as well [24,25]. Holtman and colleagues analyzed whole transcriptomes of mouse models of neurodegeneration including AD, ALS, and aging [133]. One of the main conclusions of this study was the identification of a common microglial gene core expression profile associated to disease and characterized by the expression of cell surface molecules including *Itgax*, *Axl*, *Clec7a*, and *Lgals3* (galectin-3), thus anticipating further single-cell RNAseq studies [40,41]. Importantly, four hub genes unique to this gene core expression profile were identified including *Lgals3*, *Igf1*, *Csf1*, and *Axl*, which arose as instrumental for key microglial functions under disease conditions [133]. In a recent study, Monasor and colleagues took advantage of label-free data-independent quantitation to analyze the microglial proteome in mouse models of amyloidosis at different stages of plaque deposition [155]. They identified a set of microglial Aβ response proteins (MARPs) that were upregulated at early stages of Aβ pathology and remained altered later on [155]. Top early MARPS included galectin-3, ITGAX, APOE, CLEC7A, and CD68, thus highlighting the potential instrumental role of galectin-3 in driving Aβ-associated microglia immune responses. Galectins are soluble proteins characterized by the presence of a carbohydrate-recognition domain (CRD), which bind N-acetyl-lactosamine-enriched glycoconjugates present on the cell surface or extracellular matrix [156]. This family of proteins consists of at least 15 members in vertebrates, from which galectin-3 is the only member of the chimera type [157], which is characterized by a C-terminal CRD and an N-terminal tail that allows oligomerization [156]. Within the brain, we have demonstrated the preferential expression of galectin-3 by reactive but not homeostatic microglia and to a lesser extent by reactive astrocytes [128,158], which fits well with the view that a switch from homeostatic microglia to DAM leads to upregulation of selective genes including galectin-3. Importantly, we have demonstrated the ability of microglia to upregulate and release galectin-3 in response to well-defined pro-inflammogens including LPS [159] and fibrillar Aβ [128], which are associated with different microglia polarization states [160]. The concept of DAMP has been long used in the literature and designed to define released or secreted molecules from injured neurons or other cell types [25]. More recently, the concept of neurodegeneration-associated molecular patterns (NAMPs) has been suggested to define those danger signals present under brain disease conditions triggering the appearance of the DAM phenotype [153]. Classical DAMPs have been inherently associated with binding to PRRs, including TLRs, whose activation is known to induce upregulation of pro-inflammatory genes [25]. Instead, NAMPS have been suggested to activate TREM2 to further drive the acquisition of the DAM phenotype [153]. Remarkably, we demonstrated that galectin-3 binds to and activates TLR4 [159] and TREM2 [128]. Other studies have shown that galectin-3 may also interact with insulin-like growth factor 1 (IGFR1) [161] and MER proto-oncogene, tyrosine kinase (MERTK) [162]. These pleiotropic effects make galectin-3 an attractive DAM molecule playing a central role in driving microglia-associated immune responses. Indeed, absence or inhibition of galectin-3 strongly hinders the microglia response to LPS [159], fibrillar Aβ [128], and α-synuclein (α-syn) aggregates [163]. Depletion of galectin-3 has been shown to be neuroprotective in several in vivo models of neurodegeneration including global ischemia, intranigral LPS injection, traumatic brain injury, and spinal cord injury [158,159,164]. We analyzed the role of galectin-3 in AD and found that this lectin is highly upregulated in the brains of AD patients and 5xFAD mice and particularly in plaque-associated microglia [128]. Furthermore, galectin-3 deletion in 5xFAD mice highly attenuated microglia-related immune responses, especially those related to TLR and TREM2/DAP12 signaling and decreased Aβ burden [128]. Similarly, brain levels of galectin-3 are higher in patients and mice with Huntington disease and selective galectin-3 knockdown suppressed inflammation, reduced mutant huntingtin aggregation, and improved motor dysfunction [165]. All these studies highlight the instrumental role of galectin-3 in driving deleterious disease-associated microglia polarization subtypes. 

## 3. Role of Caspases in Inflammatory Microglia Activation

Caspases play a fundamental role in the regulation of neuroinflammation as well as bearing the responsibility for the regulation and the execution of apoptosis [166], a classically viewed non-inflammatory cell death mode [167]. Caspases are an evolutionarily conserved family of cysteinyl proteases that cleave substrates after aspartate residues (for more in-depth review about caspases, see the following reviews [168,169]). Caspases were originally classified based on their function as apoptotic (caspase-2, -3, -6, -7, -8, -9, and -10) or inflammatory (caspase-1, -4, -5, -11, and -12), but it is already acknowledged that those functions are clearly overlapping in some caspases. In this part of the review we will discuss the different ways in which caspases regulate the inflammatory response.

### 3.1. Inflammasome and Pyroptosis

The innate immune response can also be triggered by intracellular inflammasomes in response to different external stimuli whose consequence is the production and release of pro-inflammatory cytokines IL-1β and IL-18 [170,171] (see Figure 1). The inflammasome is a multiprotein complex formed by a sensor protein, an adaptor protein termed ASC (apoptosis-associated speck-like protein containing a caspase activation and recruitment domains (CARD)), and caspase-1. Inflammasome sensor proteins include the cytosolic NLR family members, such as NLR Family Pyrin Domain Containing 1 (NLRP1), Neuronal apoptosis inhibitory protein(NAIP)/ NLR containing a CARD(NLRC) 4 (), NLRP3 and NLRP6 among others [172]. The NLRP3 inflammasome is one of the most extensively studied inflammasomes and is capable to sense a variety of stimuli, like bacterial toxins, and different types of bacterially derived RNA among other stimuli [173,174,175]. Activation of the canonical NLRP3 inflammasome is a process divided in two stages. First, a ‘priming’ stage triggered by the activation of TLRs induces the expression of NLRP3, pro-IL-1β, and pro-IL-18 in an NF-κB-dependent manner. Later, in the second stage, a different stimulus such as adenosine tri-phosphate (ATP) promotes K^+^ efflux, the activation of NLRP3, and the recruitment of ASC and procaspase-1, resulting in ASC oligomerization into a macromolecular aggregate, known as the ASC speck [176]. In this complex, caspase-1 is activated and cleaves pro-IL-1β and pro-IL-18 into their mature forms IL-1β and IL-18, prior to their release from the cell. Caspase-1 activation also cleaves gasdermin-D, triggering pyroptosis, a cell death mode in which plasma membrane pore formation causes cell lysis [177]. Pyroptosis is the main mechanism for IL-1β and IL-18 release [178,179], but also for release of DAMPs, such as high-mobility group box protein 1 (HMGB1) and IL-1α [180]. The noncanonical inflammasomes comprise other pathways that result in IL-1β processing, such as that involving the activation of murine caspase-11, and its human homologs, caspase-4 and caspase-5, in response to cytosolic LPS in macrophages [181], or a recently described pathway promoting activation of caspase-8 found in microglia in response to LPS as well [182]. Similar to caspase-1, caspase-11 induces pyroptosis by activating gasdermin-D as well [177,183]. 

### 3.2. Necroptosis

The binding of ligands to the death receptors TNFR, FAS, and TNF-related apoptosis-inducing ligand receptor trigger the extrinsic apoptotic pathway, but, under certain circumstances, stimulation of these receptors induces a different cell death mode, a form of programmed necrotic cell death called necroptosis. During necroptosis, cytoplasmic swelling and rupture of the plasma membrane allows the release of DAMPs, as occurs in pyroptosis [184,185]. For this reason, necroptosis can also induce an inflammatory response.

The best studied model of necroptosis is induced by TNFα that we will describe briefly (for more in-depth review about necroptosis see [186]). In the CNS, TNFR1 is expressed most prominently by microglia, astrocytes, and oligodendrocytes. The intracellular domain of TNFR1 includes a dead domain (DD) and, after stimulation of TNFR1 by TNFα leads to the formation of the membrane-associated, intracellular complex I, through the recruitment of two DD-containing proteins RIPK1 and TNFR1-associated dead domain protein (TRADD), via homotypic interaction with the TNFR1 DD. [187] In complex I, RIPK1 is subject to extensive post-translational modifications that will impact on its activity and dictates downstream cell death or survival [188]. TRADD is also able to recruit the adaptor proteins TRAF2 or TRAF5 and the cellular inhibitors of apoptosis (cIAPs), cIAP1 or cIAP2, which catalyze the ubiquitination of RIPK1 [189]. This gives RIPK1 a scaffolding function that contributes to activation of the NF-κB pathway leading to transcription of pro-survival factors, such as cellular associated death domain protein (FADD)-like interleukin-1-converting enzyme (FLICE)-inhibitory protein (cFLIP), as well as pro-inflammatory cytokines [190]. Conversely, deubiquitination of RIPK1, mediated by cIAP inhibition or the deubiquitylases, A20 or cylindromatosis, induces endosomal internalization of complex I with dissociation of RIPK1 from the plasma membrane and the recruitment of FADD and procaspase-8, resulting in the formation of complex IIa [191,192,193]. In this complex, caspase-8 can be activated promoting apoptosis and actively inhibiting necroptosis by direct cleavage of RIPK1 and RIPK3. By contrast, in the absence of caspase-8 following genetic ablation, pharmacological inhibition, or as a result of certain viral infections, RIPK1 and RIPK3 are stabilized and recruit mixed-lineage kinase domain-like protein (MLKL) into complex IIb, also known as necrosome, which initiates necroptosis [194,195]. RIPK3 mediates phosphorylation of MLKL that will lead to disruption of the cell membrane and cell lysis [196,197]. Additionally, depletion of cIAPs concurrently with TLR stimulation has also been shown to induce the spontaneous formation of the complex of RIPK1 with caspase-8 and FADD, mediated by homotypic interaction of their respective dead domain [198,199]. This complex, termed ripoptosome, can also activate RIPK3 and induce necroptosis. 

Caspase-8 is normally under the control of various isoforms of cFLIP, forming heterodimers with caspase-8. Long (cFLIP_L_)–caspase-8 heterodimers have partial enzymatic activity, leading to incomplete cleavage of caspase-8 [200,201] and, therefore, preventing its apoptotic activity; furthermore, the caspase-8 cleavage activity on RIPK1 prevents stable RIPK1–RIPK3 complex formation and necroptosis [202]. In contrast, whenever formation of the caspase-8–cFLIP_S_ heterodimer is favored, RIPK1 cleavage is blocked, and RIPK1-dependent necroptosis is, therefore, unmasked in response to loss of cIAPs [198]. On the other hand, increased expression of cFLIP_L_ and RIPK1 specifically in microglia has been shown in brain patients of multiple sclerosis. Restricted activation of caspase-8 by cFLIP_L_ preserved the pro-inflammatory capacity of RIPK1. As inflammatory microglia release TNFα, this provides an explanation for TNFα-induced necroptosis of oligodendrocytes and degeneration in cortical areas [203]. Increased levels of RIPK1 were also found in AD patients compared with controls and positively correlate with Braak stage [204,205]. Of the RIPK1-expressing cells identified, a considerable fraction were microglia. Additionally, in APP/PS1 mice, increased RIPK1 was found in microglia around Aβ plaques [205].

Increased levels of ROS were initially thought to be a leading cause of necroptosis involving the key players c-Jun N-terminal kinases (JNK) signaling [206], NOX1 [207], and aberrant mitochondrial metabolism [208]. However, subsequent studies demonstrated that, although the depletion of mitochondria from cells by enforced mitophagy prevented ROS production, it had little effect on the execution of necroptosis [209]. Nevertheless, at least in some cell types, mitochondrial ROS facilitates the initiation of necroptosis by promoting RIPK1 autophosphorylation, leading to its activation and formation of the necrosome [210,211]. In a positive feedback loop, RIPK3 kinase activates the pyruvate dehydrogenase complex, leading to enhanced aerobic respiration and associated increased ROS generation [212]. This can accelerate neurotoxicity as ROS is a key player in pro-inflammatory microglial-induced damage [25,213]. 

Necroptosis can also occur in conditions of TLR stimulation and inhibition of caspase-8 activity (see Figure 1). This has been observed in several cell types, including microglia, and involves a TRIF-dependent pathway responsible for executing necroptosis [214], [215]. As previously stated, TRIF can interact with RIPK1 and RIPK3 [102], and activation of TLR3 and TLR4 in the presence of the pan-caspase inhibitor Z- Val-Ala-Asp(OMe)(VAD)-fluoro-methyl ketone (fmk) in macrophages induces the formation of the TRIF/RIPK3 complex that leads to necroptosis [216]. Furthermore, in both microglial-based studies, although necroptotic cells released the pro-inflammatory mediator TNFα, necroptosis was TNFα-independent. Paradoxically, Fricker et al. showed necroptosis of microglia conferred in vitro neuroprotection in a co-culture setup with primary neurons with TNFα levels elevated in the necroptotic condition [214]. Similarly, in a demyelination in vivo mouse model, necroptosis of pro-inflammatory microglia was followed by repopulation of positively regulated type-I interferon microglia which promoted remyelination [217]. As far as we know, only one study has clearly attributed to necroptotic microglia a direct role in neurodegeneration, in this case in retinal cells [218]. 

### 3.3. Other Roles for Caspases During the Inflammatory Response

Apart from caspase involvement in the inflammasome, pyroptosis, and necroptosis, there are other roles where caspases have been shown to regulate the pro-inflammatory response in microglia cells. Our group described that, upon a pro-inflammatory stimulus, caspase-3/7/8 became active and were able to regulate the microglia inflammatory response without triggering cell death [159,219]. We uncovered that, upon TLR4 stimulation, a sequential activation of caspase-8 and caspase-3/7 occurred which regulated the microglial pro-inflammatory response. Subsequently, active caspase-3/7 cleaved and activated protein kinase C-δ (PKC-δ), which ultimately led to the translocation of p65 NF-κB subunit into the nucleus, inducing pro-inflammatory factors such as NOS2, TNFα, and IL-1β [219] (Figure 1). One of the interesting findings from this paper was that the caspase-3 activation was relatively small (compared with the activation that occurs during apoptosis) and it was located only in the cytosol. Both factors were key to prevent caspase-3 from triggering cell death in microglia cells. Following this, we identified cIAP2 as the mechanism behind the controlled caspase-3 activation [220]. Besides being ubiquitin ligases, cIAPs play a major role in regulating caspase activation [221]. Upon LPS treatment, the expression of microglial cIAP2 is upregulated, preventing the processing of caspase-3 into a fully activated form, thereby blocking the caspase-3 apoptotic-related activity [220]. Further studies performed by us and others observed caspase-3 activation linked to the control of the inflammatory response in microglia under a wide range of inflammatory stimuli (for detailed review, see [13]). Altogether, these findings led us to suggest that caspase-3 might work as a modulator that controls microglial activation states in response to various stimuli. In another study, we verified, using transgenic mice lacking caspase-8 in the myeloid lineage, a decreased inflammatory response in two different neuroinflammatory models (intranigral LPS and intraperitoneal 1-methyl-4-phenyl-1,2,3,6-tetrahydropyridine (MPTP) injection) with no evidence of microglial cell death [222].

In addition, the role of caspase-8 as an inflammatory inducer has been clearly established in bone marrow-derived macrophages and dendritic cells by regulating IL-1β levels. Caspase-8 was demonstrated to contribute to inflammatory functions by driving maturation of IL-1β in several studies [223,224,225,226,227]. Further to these studies, Gurung and colleagues demonstrated that FADD and caspase-8 played a crucial role in canonical and noncanonical inflammasome activation and pyroptosis [228]. Recent studies using immunofluorescence and super-resolution microscopy have also enabled the direct visualization of caspase-1 and -8 within the endogenous ASC speck structure [227,229]. Recently, as previously stated, the involvement of caspase-8 in a noncanonical NLRP3 inflammasome in microglia has been shown by its participation in IL-1β production in a caspase-1-independent manner [182]. Adding more complexity to immune cell function, necroptosis and inflammation can be molecularly interconnected processes. In some cellular models, the release of DAMPs is not the only mechanism by which RIPKs can induce inflammation as recent evidence indicates an additional role for the necrosome in the direct production of pro-inflammatory cytokines. Several lines of evidence suggest that RIPK3-MLKL signaling also activates the NLRP3 inflammasome. In macrophages, upon conditions of caspase-8 inhibition and IAP loss, RIPK3 and MLKL are required for TLR4-TRIF-induced NLRP3 activation [230]. A similar result was observed under TLR3-TRIF stimulation [231]. 

Several studies have also linked intrinsic apoptosis and mitochondrial molecules to NLRP3 inflammasome activation, although these connections are not without controversy [232]. Interestingly, mitochondrial outer membrane permeabilization (MOMP) results in cIAP degradation, promoting caspase-8-mediated processing of IL-1β [233]. Besides this, the NLRP3 inflammasome can also be activated downstream of MOMP, causing caspase-1-dependent IL-1β maturation [233]. It has already been proven that mitochondrial DNA release causes activation of cyclic GMP–AMP synthase–stimulator of interferon genes’ signaling [234]. These results further support the concept of mitochondrial apoptosis as a source of inflammatory signaling, challenging the view of apoptosis as an immunologically silent process.

## 4. Novel Aspects of the Microglia Pro-Inflammatory Response

In recent years, novel aspects of the microglia pro-inflammatory response have emerged. Here, we highlight two research areas, exosome intracellular communication and circadian rhythm transcriptional control, which can influence the microglia pro-inflammatory response.

### 4.1. Microglia Communication via Extracellular Vesicles

Extracellular vesicles are a term used to describe cell-derived membranous vesicles encompassing exosomes, microvesicles, and apoptotic bodies [235]. Exosomes (30–150 nm) are formed by plasma membrane invagination and transport of the vesicle to the early endosome. The content of extracellular vesicles is varied and most commonly consists of RNA molecules and cytosolic proteins. Exosomes containing microRNAs (miR) can affect gene expression in the recipient cell. Protein material, including toxic misfolded proteins, have been implicated in the pathogenic spread of neurodegenerative disease [236], although the role of microglia in this pathogenic spread is less well studied. However, an important role for microglia in clearance of Aβ-loaded exosomes, originating from neurons, has been demonstrated [237]. Microglia can propagate a neuroinflammatory signal using exosomes [236]. Although exosomes can fuse directly with outer membrane of the recipient cell, releasing their contents directly into the cytoplasm [238], endocytosis of exosomes by the recipient cell is the most common uptake mechanism [239,240,241,242]. Specifically, microglia themselves take up exosomes by micropinocytosis [240]. Exosomes can be released by homeostatic microglia and influence CNS events such as synaptic transmission [243,244]. However, in response to pro-inflammatory stimuli, microglia increase shedding of extracellular vesicles containing pro-inflammatory cytokines and miRNAs that promote neuroinflammation. LPS-stimulated microglial extracellular vesicles contain IL-1β and miR-155, both contributing to pro-inflammatory signaling [245,246]. Exosomes from BV2 microglia treated with α-syn contain TNFα and MHC Class II proteins and result in increased neuronal apoptosis [247].

Exosomes progress through the endosome maturation process, which themselves accumulate additional intraluminal vesicles, eventually fusing with the cell’s plasma membrane, and releasing of the vesicle contents, including exosomes into the extracellular space [248,249]. It is not surprising, due to the phagocytic nature of microglia, that vesicles in the extracellular environment are preferentially endocytosed by microglia and can affect their phenotype. In an in vitro model of ALS, exosomes from NSC-34 motor neuron-like cells transfected with SOD1(G93A) contained miR-124, which induced a strong pro-inflammatory microglia phenotype characterized by NF-κB-driven expression of pro-inflammatory cytokines and generation of NO [250]. In a mouse model of traumatic brain injury (TBI), extracellular vesicles were purified from injured brain regions and neuronally released extracellular vesicles were found to contain miR-21. The authors suggested that the neuronally derived exosomes increased concurrently with the appearance of activated microglia and hypothesized that exosomes may be taken up by the surrounding microglia with implications for their phenotype [251]. 

### 4.2. Circadian Rhythm Regulates Microglia Pro-Inflammatory Response

Circadian rhythms affect a range of biological processes including the inflammatory response [252]. Circadian stimulus regulators, known as zeitgebers, include the light–dark cycle, food intake, physical activity, and change of temperature. Neurons of the suprachiasmatic nucleus (SCN) receive zeitgeber signals and that signal is relayed to peripheral oscillators within various organs. Genes with E-box promoters known as Circadian Locomotor Output Cycles Kaput (CLOCK)-controlled genes (CCG) are transcribed by heterodimerization of transcription factors aryl hydrocarbon receptor nuclear translocator-like (ARNTL) with CLOCK. Included among CCG are *PER1-3*, *CYR1-2*, *NR1D1* (REV-ERBα), and *NR1D2* (REV-ERBβ), which deactivate the CLOCK transcription factor heterodimers, thereby creating a feedback inhibition and concluding the circadian cycle [252]. 

Chronic exposure of mice to dim light at night increased microglia-mediated cytokine levels after LPS exposure compared with unexposed mice [253]. This implied that disruption of circadian rhythms can adversely affect the immune response and particularly the microglia pro-inflammatory response. Microglia isolated from rat hippocampi at various stages of the light–dark cycle expressed pro-inflammatory cytokines in a circadian pattern, with the highest expression peaking midway through the light cycle [254]. Microglia isolated from the light phase were more reactive to LPS stimulus compared to dark phase-isolated microglia. Indeed, a follow up study provided evidence that dysregulation of neuroinflammation is linked to a disrupted circadian rhythm in aged animals [255]. Specifically, microglia isolated from aged rats lost the circadian pattern of pro-inflammatory cytokine expression observed in younger rats, with aged rats retaining the high levels of TNFα and IL-1β normally only seen in the light phase of young rats. 

A direct link between the circadian rhythm and microglia pro-inflammatory phenotype comes from a knockout mouse study, where genetic deletion of REV-ERBα was sufficient to activate hippocampal microglia and increase pro-inflammatory gene transcription [256]. When subjected to peripheral LPS injection, the pro-inflammatory response of REV-ERBα knockout mice was enhanced. Mechanistically, a CHiPseq experiment revealed that REV-ERBα binds the promoter of NF-κB target genes linked to the inflammatory response such as *Traf2* and NF-κB inhibitor beta (*Nfkbib*), an inhibitor of NF-κB nuclear translocation. A follow-up study in a 5XFAD mouse model of AD revealed that inhibition of REV-ERBα pharmacologically or through genetic knockdown pushed microglia towards a phagocytic phenotype as measured by an increase in Aβ clearance and a decrease in plaque deposition [257].

## 5. Epigenetic Control of the Microglial Pro-Inflammatory Response

**Figure 2:**
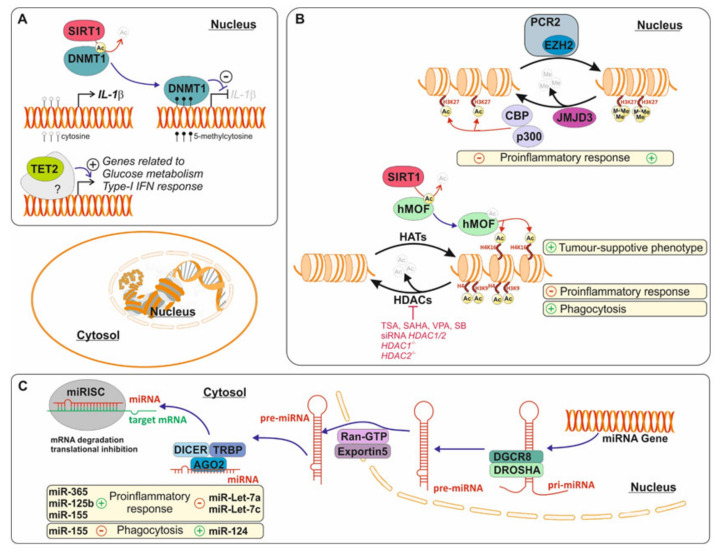
Schematic representation of epigenetic control of the inflammatory response in microglia. (**A**) SIRT1 deacetylates and activates DNMT1, provoking IL-1β repression through DNA methylation. TET2 regulates the expression of genes related to type I interferon response and glucose metabolism independently of its enzymatic activity, most likely acting as a scaffold for other proteins in gene promoter regions. (**B**) The histone methylase EZH2 and the histone demethylase JMJD3 increase or decrease the pro-inflammatory response respectively through H3K27 methylation/demethylation (once H3K27 is demethylated it becomes acetylated by CBP/P300). The use of HDACi such as TSA, VPA, SAHA, or SB, or the decrease in expression in HDAC1 and HDAC2 decreases the pro-inflammatory response and increases microglial phagocytic capacity. SIRT1 interacts with hMOF, resulting in the deacetylation and activation of hMOF, which then acetylates H4K16 and promotes the tumor-supportive microglial phenotype. (**C**) MiRs regulate the microglial inflammatory response. MiRs are transcribed and processed in the nucleus into pri-miR and pre-miR, which are then translocated to the cytosol with the aid of Ran-GTP/exportin 5. Once in the cytosol, pre-miR becomes miR through processing mediated by DICER/TRBP/AGO2. Finally, miR targets mRNA, forming a miRISC complex, which provokes either mRNA degradation or inhibits translation of the targeted genes. MiR-365, miR-125b and miR155 promote a pro-inflammatory response while miR-Let7a and miR-Let7c inhibit it. Also, miR-155 inhibits phagocytosis while miR-124 promotes it.

Changes in the microglia environment trigger various epigenetic events, including DNA methylation, histone modifications, and miRs [258], which can modify the microglia inflammatory response and contribute to the progression of several neurodegenerative diseases [259] (Figure 2). Although there are numerous epigenetic events that can modulate multiple gene expression, we will only discuss those related to the pro-inflammatory response in microglia cells and that may initiate or contribute to the progression of neurodegenerative diseases.

### 5.1. DNA Methylation during the Microglia Pro-Inflammation Response

DNA methylation is a chemical modification that typically results in gene repression and is characterized by the addition of a methyl group on cytosines of CpG sites located close to mammalian promoters or regulatory regions forming 5-methylcytosine (5-mC) [260]. Gene methylation is regulated by two families of enzymes with opposing activities, the DNA methyltransferases (DNMTs) and the ten-eleven translocation methylcytosine dioxygenases (TETs) [260]. While DNMTs are responsible for adding the methyl group and repressing gene expression, gene demethylation is mediated by the TET family of enzymes that oxidize 5-mC to 5-hydroxymethylcytosine (5-hmC) and promote gene expression [260].

Initial epigenetic studies in the field of neuroinflammation concerning DNA methylation were mostly descriptive, focusing on measuring differences in global DNA methylation levels in microglia under various conditions. For instance, global hypomethylation was observed in a specific microglia population in a TBI rat model [261]. The authors correlated this hypomethylation with control of the inflammatory response. Later, as studies became more mechanistic, direct links were made between changes in DNA methylation and the expression of pro-inflammatory factors. For instance, changes in the neonatal handling of rats provoked an altered DNA methylation pattern in microglia that affected the levels of IL-10 and changed rat behavior in a morphine-induced glial activation model [178].

Several studies have shown how the expression of IL-1β, a key cytokine in the pro-inflammatory response, is affected by DNA methylation. Chemical inhibition of DNA methylation (using 5-azacytidine) increases IL-1β expression [262]. A separate study confirmed that DNMT1 was responsible for IL-1β gene repression [263]. Interestingly, in this same study, the authors suggest that the mechanism regulating DNMT1 activity depends on its interaction with Sirtuin 1 (SIRT1), a histone deacetylase. The study also demonstrated that deacetylated DNMT1 is active and can repress IL-1β expression and that deficiency of SIRT1 leads to aging- or tau-mediated memory deficits in hTau-P301S mice via inhibition of DNMT1 and IL-1β upregulation [263]. 

The TET family, 5mC dioxygenases, regulates inflammation in several peripheral immune cell types [264,265]. A recent study from our lab showed, for the first time, that TET2 regulates the pro-inflammatory response in microglia [259]. We described how TET2 is required for the full pro-inflammatory response upon TLR4 stimulation including the LPS-induced expression of IL-1β, IL-6, and TNFα [259,266]. In this case, TET2 regulates the inflammatory response independently of its enzymatic activity, acting more as a scaffold for other proteins as has been shown previously in other cell types such as peripheral macrophages [267].

Notably, the relevance of TET2 in neurodegenerative diseases has come to attention in a recent paper published by Cochran and colleagues [268]. In this paper, the authors established an association between TET2 mutations and an increased disease risk for AD, frontotemporal dementia, and ALS using genome sequencing. The authors could not pinpoint which cells bearing these TET2 mutations were responsible for the increased disease risk but, based on our publication, we suggest that microglial TET2 could be the culprit. In our study [259], we observed an increase in the expression of microglial TET2 in close proximity with β-plaques in 5xFAD mice. Nevertheless, it cannot be discarded that neuronal TET2 mutations could also be the reason for increased neurodegenerative disease risk or even a combined effect in microglia and neurons. More research using transgenic mice lacking TET2 in either neurons or microglia would be necessary to clarify this. 

### 5.2. Histone Modification and Microglia Activation

Histone are proteins that allow nuclear DNA to supercoil, creating a compacted structure called nucleosomes [269,270,271]. Histones may undergo different types of post-translational modifications, which will alter their binding to DNA. These modifications include acetylation, phosphorylation, methylation, ubiquitination, sumoylation, and ADP-ribosylation [271] but for this review we focused on those involved in the control of microglial immune response.

Histone acetylation on lysine residues was one of the first post-translational modifications linked to the inflammatory response in epithelial cells and peripheral macrophages [272,273]. Histone acetylation is associated to transcriptionally active regions (or euchromatin) and its levels are regulated by histone deacetylases (HDACs) and histone acetyltransferases (HATs) [274]. We can distinguish four families of HATs and four families of HDACs (classes I–IV) [270]. The development of HDAC inhibitors (HDACi) have allowed researchers to investigate the contribution of histone acetylation to numerous pathological conditions including cancers [275].

Microglia cells treated with LPS induce the expression of all HDACs [276], anticipating a role of these proteins during the inflammatory response. Further studies using various HDACi confirmed the importance of HDACs in the regulation of the microglial inflammatory response. For instance, nonspecific HDACi, such as trichostatin A (TSA), vorinostat (SAHA) (both inhibitors of classes I and II HDACs), or valproic acid (VPA) (inhibitor of class I HDACs), suppress the pro-inflammatory response after treatment with TLR3 or TLR4 agonists in vitro [276,277]. Similar anti-inflammatory effects have also been observed using siRNA against HDAC1 and HDAC2 or selective HDACi for HDACs 1, 2, and 3 (apicidin, MS-275, and MS-192) in BV2 cells treated with LPS [278]. Likewise, in vivo treatment with TSA or sodium butyrate (SB) (another classes I and II HDAC inhibitor) prevents microglia activation and confers neuroprotection in a lipopolysaccharide-sensitized neonatal hypoxic-ischemic brain injury and an ischemic-stroke model [279,280]. In a separate study focusing on the microglial response in 5xFAD mice, the authors observed that the depletion of HDAC1 and HDAC2 in microglia significantly increased the expression of phagocytosis genes, enhancing microglial amyloid phagocytosis, decreasing amyloid load, and improving cognitive impairment [281]. Based on these findings, the use of different HDACi as potential treatments for neurodegenerative diseases has been suggested [282]. However, one should be aware of the long-term off-target effects that the use of nonspecific HDACi could have over other cell populations [282]. 

Which histones and lysine residues are targets of HDACi during microglial neuroinflammatory response? There are several studies that address this. For instance, SB promotes enrichment of histone 3-lysine 9-acetylation (H3K9ac) in microglia upon ischemia [280]. This enrichment in H3K9ac decreases the expression of *Tnf* and *Nos2* and increases *Il10* gene expression. Genome-wide profiling of histone H3K9ac and H3K27ac in healthy animals revealed that the global levels of these residues are not significantly affected in microglia lacking HDAC1 and HDAC2 [281] in wild-type (WT) mice. However, double knockout for both HDACs in the 5xFAD model provoked an enrichment of H3K27ac in the promoter of *ApoE* and *Il33*, while H3K9ac was decreased in the promoter of IL-4 but increased in the promoter region for chemokine (C-C motif) receptor 3 (*Ccr3*) [281]. Depletion of HDAC1/2 or chemical inhibition using selective HDAC inhibitors for HDACs1–3 shows an increase in acetylation of H4, through the authors did not specify which residue was affected [278].

SIRT1, a member of HDAC class III, also modulates the microglial response. We discussed previously how SIRT1 promotes activation of DNMT1 and how this affects the expression of IL-1β [263]. Interestingly, SIRT1 can also interact with the HAT hMOF, in the nucleus, facilitating the hMOF-mediated increase in H4K16 acetylation at specific promoters of genes related to a tumor-supportive phenotype in glioma [283].

Another post-translational modification linked to the inflammatory response is histone methylation on lysine residues. This process is carried out by histone methylases and demethylases [284]. In microglia cells, inhibition of histone H3K27me3 demethylase Jumonji domain containing 3 (*Jmjd3*) causes microglial activation and neuronal cell death both in vitro and in a MPTP mouse model. JMJD3 is necessary for the microglial anti-inflammatory response and its depletion promotes over-activation of the pro-inflammatory response [285]. Histone methyltransferase enhancer of zeste homolog 2 (EZH2) is known to reverse the effect of JMJD3 by methylating histone H3K27. In fact, in activated microglia, EZH2 gene expression is found to be significantly and rapidly increased upon pro-inflammatory stimuli and mediates TLR-induced pro-inflammatory gene expression [286,287]. These effects include the transcription of *Irf1*, *Irf8*, and *Stat1*, all key drivers of microglia activation under neuroinflammatory conditions [288]. Depletion of EZH2 or selective inhibition using small-molecule inhibitors EPZ-6438, GSK343, or GSK126 suppressed the expression of pro-inflammatory genes [286,287] and diminished macrophage/microglial activation and autoimmune inflammation in dextran sulfate sodium-induced colitis and experimental autoimmune encephalomyelitis [287].

### 5.3. MicroRNAs and Pro-Inflammatory Microglia

MiRs are small, noncoding RNAs of 19–25 nucleotides in length, regulating many signaling pathways under physiological and disease conditions [287]. Here we will highlight examples of miRs known to modulate the microglia response. For instance, microglial activation in the SOD1G93A ALS mouse model is associated with the overexpression of several miRs, such as miR-22-3p, miR-125b-5p, miR-146b-5p, miR-155-5p, miR-214-3p, and miR-365-3p [289]. MiR-365 and miR-125b induce TNFα transcription and modulate the expression of genes involved in the IL-6 and STAT3 signaling pathway [290]. The miR-155 is increased and promotes the pro-inflammatory response in vitro upon LPS stimulus [291] and has been linked to microglial activation in ALS mouse models [292,293]. Interestingly in ALS, the over-expression of miR155 decreases microglial phagocytic activity towards dead neurons. Once ablated, microglia recover their phagocytic activity and the survival of SOD1(G93A) ALS mice is prolonged, making miR-155 a possible therapeutic target for ALS [292,293]. MiR-155 is not alone in regulating microglia phagocytosis. MiR-124 promotes microglial phagocytic activity in rodent models of spinal cord contusion injury [294]. The miR-Let-7 family similarly regulates microglia activation. Microglia with high levels of miR-Let-7a decrease the production of nitrite and the expression of NOS2 and IL-6 and increase the expression of IL-4, IL-10, and brain-derived neurotrophic factor (BDNF) [295]. MiR-let-7c-5p modulates microglial activation upon trauma [296,297] by inhibiting the caspase-3-mediated pro-inflammatory response through targeting caspase-3 expression.

## 6. Immunometabolism—The Link between Small Molecules and the Immune Response

Over the past decade, immunometabolism has emerged as an exciting research field, mainly due to significant developments of new, sensitive technology such as metabolomics and other system-based biology applications [298]. Immunometabolism is defined as a specific collection of metabolic pathways with regulatory functions related to immunity and the immune response [299]. This appealing line of research meant rediscovering cellular metabolism at its core, pinpointing immunometabolites that exert regulatory functions, and command intracellular pathways during immune cell activation [300]. An immunometabolite is thus defined as molecule, which has a profound influence on how immune cells both respond to and control their environment. 

Recently it has been demonstrated that dysregulation of energy metabolism in microglia, even minor alterations, relate to the progression of neurodegenerative disorders such as AD and PD [301,302,303]. As such, microglia can adapt and alternate their use of metabolites for the generation of energy according to their current need [304,305]. These publications centered on microglia and neurodegenerative disorders highlight how a mechanistic understanding of microglia immunometabolism is of profound interest to understand both onset and progression of AD. Further investigations into metabolism and its connections to neurodegenerative disease are still required as such knowledge may lead to treatments that are more effective [41,306]. 

**Figure 3:**
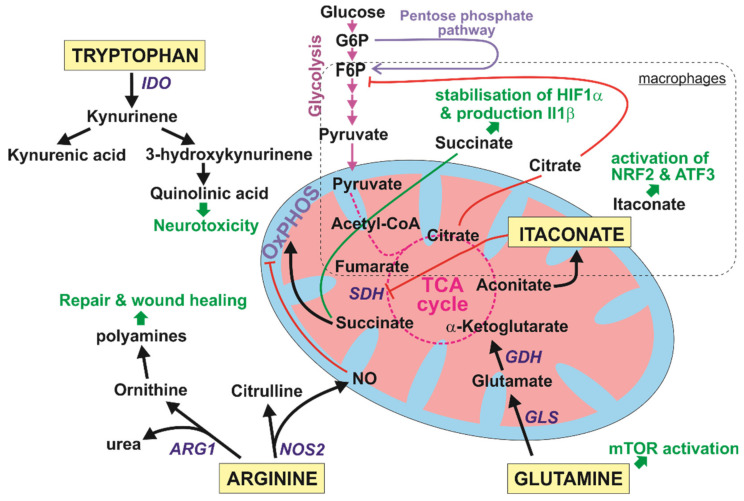
Overview of metabolic alterations in microglia metabolic pathways associated with the four immunometabolites (yellow boxes): Itaconate, tryptophan, arginine, and glutamine in microglia. Metabolic changes in itaconate observed in macrophages and the TCA cycle (dotted line) include activation of NRF2/ATF3, inhibition of SDH as well as stabilization of HIF1α, production of IL-1β, and increased OxPHOS through succinate accumulation as well as inhibition of glycolysis through citrate. Tryptophan catabolism results in the conversion of tryptophan to kynurinene by IDO and the production of kynurenic acid or 3-hydroxykynurinene, the latter of which produces quinolinic acid with neurotoxic effects. Arginine catabolism is illustrated by either the generation of ornithine by ARG1, ultimately leading to polyamine production used for repair and wound healing, or production of citrulline and NO by NOS2. NO effectively inhibits OxPHOS. Glutamine results in mTOR activation and is converted to glutamate through GLS. Glutamate is further metabolized by GDH to form one of the principal components of the TCA cycle: α-ketoglutarate.

In this part of the review, we aimed to highlight important findings concerning microglia immunometabolism; macrophage-related research is cited and discussed in cases where there is a clear lack of data regarding microglia. Of importance is a set of immunometabolites included in several interlinked metabolic processes: The phenotypic energy switch and amino acid catabolism (arginine, tryptophan, and glutamine), metabolic changes that enable microglia cells to elicit their immune functions (illustrated in summary in Figure 3). It is, however, important to state that even though some metabolic alterations observed in microglia mimic those observed in other immune cells (e.g., macrophages), their metabolism is based upon the unique conditions encountered in the brain, thus, in some sense, painting a different picture of immune response [307,308]. 

### 6.1. Bioenergetics of Microglia: A Phenotypic Energy Switch

The tricarboxylic acid (TCA) cycle serves as the main energy hub in cellular metabolism and, thus, sits in the very center of metabolic reprogramming mechanisms related to immune responses. The TCA cycle relates directly to oxidative phosphorylation (OxPhos) and generation of ROS. The TCA cycle starts by converting pyruvate, generated from the pentose phosphate pathway, glycolysis, or glycogen metabolism into acetyl-CoA, which then enters the TCA cycle [298]. Macrophage-based experiments and, to some extent, microglia-oriented research established that myeloid cells are able to switch their energy needs from an aerobic profile based on OxPhos and fatty acid oxidation (FAO) to a profile based on anaerobic profile utilizing glycolysis and the pentose phosphate pathway when exposed to pro-inflammatory stimuli [309,310,311,312]. In macrophages, several breakage points in the TCA cycle result in a significant switch in bioenergetics that allows for the signaling molecules itaconate, succinate, and citrate to escape from the mitochondria [313,314,315]. In the context of microglia, as we were not able to find any substantial evidence related to itaconate, the so-called “posterchild of metabolic reprogramming” [300], we describe findings in macrophages. Briefly, the generation of itaconate starts by conversion of citrate to aconitate, which departs away from the TCA cycle during inflammatory stimuli to form itaconate. Itaconate inhibits succinate dehydrogenase (SDH) as well as inducing the anti-inflammatory proteins nuclear factor erythroid 2-related factor 2 (NRF2) and transcription factor 3 (ATF3). NRF2 fills the role as an oxidative stress sensor and is regulated by Kelch-like ECH-associated protein 1 (KEAP1), a protein which targets NRF2 for proteosomal degradation [316]. As a response to oxidative stress, itaconate inactivates KEAP1, which results in the release of NRF2 that induces transcription of numerous NRF2-related genes serving as a shield against the cytotoxic effects of oxidative stress [310]. Moreover, itaconate may induce ATF3, which serves as a negative regulator of immune activation related to the regulation of cytokines including IL-6 and IL-12 as well as a regulator of mitochondrial stress [317].

Past research has shown that SDH is responsible for driving cellular metabolism towards the generation of ROS, thus connecting the metabolite itaconate through its inhibition of the enzymatic activity of SDH by blocking its active site, to oxidative stress [311]. 

Furthermore, the inhibition of SDH leads to an accumulation of succinate, which acts as a signal to stabilize hypoxia inducible factor 1α (HIF-1α). HIF-1α specifically controls gene expression of IL-1β and other HIF-1α-dependent genes that regulate the expression of enzymes in the glycolysis pathway [300,313]. Accumulated citrate, on the other hand, is transported out of the mitochondria and utilized for synthesis of fatty acids, generation of NO, and prostaglandins [318]. In a recent study by Cordes and colleagues, exogenous itaconate treatment was tested in primary rat cortical neurons and astrocytes showing that itaconate acts as a mitochondrial regulator that controls redox metabolism and further connects to glutathione metabolism [311]. Nevertheless, the findings concerning itaconate and its significant regulatory roles in macrophages call out for further investigation in microglia in order to understand if similar regulatory functions related to energy metabolism occur and may be a potential target in microglia.

### 6.2. Amino Acid Metabolism and Immune Functions of mTOR

The mTOR (mammalian target of rapamycin) is a serine/threonine protein kinase that regulates vital cellular processes as well as functioning as a sensor of amino acid (AA) levels. The two complexes of mTOR (mTORC1, mTORC2) sense and integrate various metabolic signals but have different functions, each of which possess unique protein machinery and phosphorylate different substrates [319,320]. Before discussing mTOR relevance in amino acid metabolism, it is noteworthy to mention the immunomodulatory effect of mTOR signaling over microglial activation. Pro-inflammatory microglia induce mTOR signaling via the PI3K/AKT signaling pathway, decreasing autophagy and increasing the cytokine immune response [321,322]. The use of rapamycin, an inhibitor of mTOR and inducer of autophagy, has been shown in several studies to dampen the microglial pro-inflammatory response in different conditions both in vitro [321,322] and in different in vivo models [323,324]. Moreover, mTOR also influences mitochondria turnover where ROS-generating mitochondria are predominantly sequestered by mitophagy [325]. Briefly, mitophagy is a selective autophagy pathway that recognizes damaged mitochondria and sequesters them into autophagosomes, thereby forming mitophagosomes that eventually fuse with lysosomes, leading to degradation [326].The role of mitophagy in pro-inflammatory microglia has been shown in several studies, where induction of mitophagy reduces neuroinflammation and AD pathogenesis [327]. Additionally, the mitophagy inducer, mitochonic acid 5 (MA-5), is reported to reduce neuroinflammation in microglia [328]. These findings suggest that induction of autophagy is linked to an anti-inflammatory phenotype; the mechanisms involved are not yet clear. Nevertheless, some research suggests that autophagy could play a role as a control mechanism to limit further mitochondrial ROS production and the subsequent microglial activation [329].

In summary, mTOR functions as a master regulator of metabolism and cell proliferation through various mechanisms by integrating signals of nutrient availability and cellular energy status to regulate protein and lipid synthesis and autophagy as well as metabolic flux. Perhaps not surprisingly, the availability of a specific set of AA and their metabolism also have specific implications for the immune cell response in fascinating ways. Thus, the sensing of extra- and intracellular AA levels by mTOR and its purpose of controlling anabolic–catabolic balance is of prime importance to understand immunometabolism and its connection to AA [330]. Here, we will highlight some of the known functions of arginine, tryptophan, and glutamine concerning microglia immunometabolism.

#### 6.2.1. Arginine: A Fork in the Road Ahead

Arginine metabolism has been reported to play a key role in immunometabolic responses in microglia [331,332,333]. The current evidence suggests two alternative and competitive directions for arginine: Into the NO synthesis pathway associated with the inflammatory response or into the arginase pathway associated with wound healing and repair [334,335]. Briefly, NO synthesis involves the conversion of arginine into citrulline by NOS2 and ultimately the production of NO [334] while arginase 1 (ARG1) converts arginine into ornithine, leading to the generation of polyamines such as spermidine and spermine, which contribute to tissue remodeling and wound healing [336]. The generation of NO also has implications for energy metabolism as it effectively inhibits OxPhos thereby causing a shift towards glycolysis [337]. Thus, it is clear that the immunometabolite arginine represents an important precursor-molecule for the microglia cellular immune response.

#### 6.2.2. Tryptophan: The Two Fates of Kynurenine

Tryptophan plays a key role in modulating immune function through its catabolism by the enzyme indoleamine-2,3-dioxygenase (IDO) into kynurenine and its downstream pathways [338]. An inflammatory response is known to stimulate IDO expression and, thus, catabolism of tryptophan to kynurenine [298]. Of importance is the plausible immunomodulatory and immunosuppressive effects that these metabolic products display [338]. The generation of kynurenine and its possible metabolic products quinolinic acid, 3-hydroxykynurenine and kynurenic acid act by different mechanisms in astrocytes and microglia (reviewed in [339]). Briefly, kynurenic acid produced by astrocytes is neuroprotective through its ability to clear glutamate spillover [340], while its counterparts, quinolinic acid and 3-hydroxykynurenine, that are produced in microglia can cause neurotoxicity by several different mechanisms [341,342]. Quinolinic acid acts as an agonist of the N-methyl-D-aspartate receptor but also through additional cytotoxic mechanisms (reviewed in detail by [343]) while 3-hydroxykynurenine generates excitotoxic effects through activation of a subunit (NR2B) of the N-methyl-D-aspartate receptor [344]. As such, the catabolism of tryptophan to kynurenine leads to products with neurotoxic properties; however, it is certain that a full understanding of tryptophan as an immunometabolite requires further examination. 

#### 6.2.3. Glutamine: The Alternative Energy Source

Expression of glutamate transporter-1 and glutamine synthetase (GS) has been demonstrated in microglia, which indicates the possibility of glutamate scavenging into glutamine as an immune response mechanism [345,346]. It is plausible that increased levels of glutamine fill a regulatory role in microglia through its activation of the mTOR pathway [347]. Furthermore, Palmieri and colleagues have demonstrated that GS inhibition enhances the inflammatory response of activated microglia in vitro, thus effecting the redox balance within the cell [348]. Glutamine may also serve as an alternative energy source supplying the TCA cycle/OxPhos as a precursor to α-ketoglutarate by actions of glutaminase (GLS) and glutamate dehydrogenase (GDH) in similar fashion as in glutamine-addicted cancer cells [349] with the goal of ensuring a well-functioning immune surveillance. Bernier and colleagues have demonstrated that glucose-deprived microglia displayed a high degree of metabolic flexibility in vivo and in vitro by utilizing a switching mechanism from glucose as energy source to glutaminolysis in an mTOR-dependent manner [330]. Glutamine as an immunometabolite demonstrates the flexibility of microglia to use alternative energy sources in order to execute their functions. 

## 7. New Tools to Study Microglia Function

Due to the sensitivity of microglia to their surrounding environment, the information generated describing their role in physiological and diseased conditions may have been compromised depending on the experimental conditions used to generate the data. A new array of tools has been developed to improve the quality of results independently of the experimental procedure used. Here, we review some of these new tools to study microglial functions. 

### 7.1. New Imaging Tools to Study Microglia

The development of in vivo two-photon microscopy and the appearance of new microglia-specific mouse reporter lines enabled the first detailed “live” in vivo studies of microglia [350,351]. These studies demonstrated that microglia are constantly surveying their microenvironment through their many ramified processes. Further studies showed that microglia survey specific neuronal elements such as synapses [352] and neuronal cell bodies [353]. Recently, Cserép and colleagues were able to describe, by means of this technology, the mechanism involved in microglia and neuronal soma interactions, uncovering the role of the microglia-specific P2Y12 purinergic receptor (P2RY12) [354], and its potential neuroprotective role upon ischemic injury. These observations led the authors to suggest microglial P2RY12 as a new potential therapeutic target in ischemia [355]. On the other hand, by means of this technique, the involvement of microglia in Aβ plaque formation in 5xFAD mice was confirmed [301]. The authors found that plaque-associated microglia take up Aβ, thus causing microglia cell death to further contribute to Aβ plaque growth.

Other brain imaging techniques linked to microglia are currently applied in early-stage preclinical or clinical research of neurodegenerative diseases. PET imaging enables in vivo quantification and visualization of physiological processes using tracer molecules labelled with positron-emitting isotopes. Despite decades of research both by academia and pharmaceutical companies into the development of treatments based on targeting the neuroinflammation network, there has been a lack of success in the development of translatable therapies [356]. For example, therapies applied at the late stages of AD seem unlikely to be effective, since ongoing chronic neuroinflammatory processes will have already caused irreversible damage [357]. In vivo imaging of biomarkers of neuroinflammation should assist in early detection of AD and other neurodegenerative diseases and help clinical researchers measure early responses to therapeutic interventions [358,359,360].

In the early stages of PET imaging studies, the use of translocator protein (TSPO), an 18-kDa outer mitochondrial membrane protein, as a radiotracer was demonstrated to be a suitable neuroinflammation biomarker. TSPO expression was observed by immunohistochemistry of microglia and PET imaging in various animal disease models such as AD, stroke, brain injury, and epilepsy [361,362,363,364,365,366]. However, translation of TSPO to the clinic was hampered by limitations in the differential sensitivity of radioligands to a single-nucleotide polymorphism in the TSPO gene [367,368]. Besides that, some studies raised a cell-specificity issue, as expression of TSPO was also found in astrocytes [369,370]. For these reasons, researchers have developed new PET radiotracers targeting other markers of neuroinflammation such as cannabinoid-2 receptor, COX2, the P2X7 purinergic receptor, and ROS [371]. Other PET targets are GSK-3, monoamine oxidase-B, and sphingosine-1-phosphate receptor 1, but these remain in a relatively preliminary stage of preclinical development. A recent study has shown the development of a new PET imaging radiotracer, [^11^C] CPPC[5 -cyano-N-(4-(4-[^11^C]methylpiperazin-1-yl)-2-(piperidin-1-yl)phenyl)furan-2-carboxamide], a positron-emitting, high-affinity ligand that is specific for the macrophage colony-stimulating factor 1 receptor (CSF1R) [372]. Within the brain, CSF1R is mainly expressed by microglia, while its expression in other cells including neurons is low [373]. CSF1R directly controls the development, survival, and maintenance of microglia and plays a pivotal role in neuroinflammation [374,375]. Horty and colleagues showed that specific binding of this radiotracer was increased in mouse and nonhuman primate models of LPS-induced neuroinflammation, murine models of AD, and in postmortem AD human brain tissue. Radiation dosimetry studies in mice demonstrated that [^11^C]CPPC is also safe for human studies. This evidence led the authors to propose CSF1R as a new biomarker to study microglia activation by means of PET imaging in brain diseases with neuroinflammatory component.

### 7.2. Human iPSCs as Source of Microglia

In vitro culture of microglia is an important platform for in-depth studies of microglia function. The vast majority of the data generated in the microglia field so far is based on animal models and in particular rodents. Though these studies have laid the foundations of microglia biology, we cannot disregard species’ differences between mouse and human models. This caveat is abundantly clear in studies of polygenic diseases such as AD [24,376,377]. In addition, recently several studies have shown key differences between rodent and human microglia at transcriptomic levels [39,378,379]. These differences could be among the reasons behind the current lack of translatable therapies in multiple diseases including neurodegenerative disorders [380].

Researchers have sought to address this caveat by developing methods to establish primary culture of human microglia. This approach has provided many important insights into human microglia, such as the differential gene expression signatures between microglia and macrophages across varying activation states [381]. However, some drawbacks limit the utilization of this approach for long-term culture studies of microglia. Namely, the difficulty in obtaining cells in adequate numbers from not very homogenous preparations and diverse patient conditions are challenges to be overcome. Furthermore, removing microglia from the brain drives transcriptomic and phenotypic changes that compromise the applicability of this primary culture model as a reliable in vivo counterpart [35,378,382]. For these reasons, new techniques have been applied to address those problems.

Pluripotent stem cell technology holds great promise for research of a broad spectrum of human diseases. Embryonic stem cells (ESCs), which are derived from the inner cell mass of blastocysts, are pluripotent cells with the ability to proliferate indefinitely and to differentiate into cells of all three germ layers: Ectoderm, mesoderm, and endoderm [383,384]. However, the isolation of human ESCs raises ethical concerns as they are derived from pre-implanted embryos and there are restrictions on their use [385]. Alternative approaches to ESCs have emerged thanks to the groundbreaking discovery of cellular reprogramming [386] and the generation of iPSCs from adult somatic cells [387,388]. The iPSCs can now be generated from human cells for a number of purposes including autologous cell therapy, development of new therapeutic drug candidates, and in vitro modelling of monogenic and multigenic human diseases [389,390,391].

Differentiation of human iPSCs into microglia can significantly contribute to the understanding of the microglial role in neurological diseases. Based on the findings of developmental ontogeny studies, the generation of microglia from iPSCs must transition through a sequence of lineage states resembling primitive hematopoietic precursor cells, MYB-independent erythromyeloid progenitors, and, ultimately, microglia within the brain [8,392,393]. Recent differentiation protocols propose a variety of factors for generation of microglia-like cells, but all attempt to recapitulate embryonic differentiation pathways [394,395,396,397,398]. In these studies, basic features of microglia, such as inflammatory responses, migration, and phagocytosis, are reconstituted in monoculture experiments of iPSC-derived microglia, but still some transcriptomic and phenotypic deficiencies persist due to the lack of appropriate interaction with cells that microglia normally encounter in the brain. To address this, various approaches have been undertaken to mimic the CNS environment in which microglia normally reside. In this sense, Abud and colleagues used a three-dimensional brain organoid (BORG) co-cultured with iPSC-derived microglia [394]. BORGs include neurons, astrocytes, and oligodendrocytes that self-organize into a cortical-like network but lack microglia. In this situation, microglia from erythromyeloid progenitors migrated, engrafted within BORGs, and were able to adopt a more ramified morphology than their purely in vitro counterparts. In a separate model from the same study, iPSC-derived microglia were grafted into the cortex of adult immuno-compromised “MITRG” mice humanized for CSF1, CSF2/IL3, and thrombopoietin. These conditions allowed the long-term survival of microglia and expression of some homeostatic markers such as P2RY12 and TMEM119 in the xenotransplanted cells. In a similar setup, the transplantation of human iPSC-hematopoietic progenitor cells in MITRG mouse pup recipients resulted in robust engraftment of cells that acquired typical microglial surface markers and morphology [399]. Importantly, transcriptomic analysis of xenotransplanted microglia performed two months after transplantation showed that these cells acquired a gene signature that closely resembled human ex vivo microglia, while correcting many of the transcriptomic deficits present in human primary culture and iPSC-derived microglia [378].

Although both the BORG and the xenotransplantation approaches allow the interaction of iPSC-derived cells with surrounding neurons, both methods have pros and cons. For instance, in the BORG approach, since microglia and BORG cells are generated from the same human iPSC line, all cells share human origin and genetic background, but they lack the architecture of the brain tissue, as well as vascularization, nutrient, and oxygen support. Meanwhile, in the xenotransplantation approach, these last issues are solved, although it must be done in humanized mouse. However, the greatest strength of this model is that it allows the direct assessment of human in vivo microglia´s role in disease animal models of neurodegeneration. For instance, in the study of Hasselmann and colleagues, xenotransplanted human microglia responded to the inflammatory stimulant LPS and even migrated and phagocytosed amyloid plaques using the 5X-FAD-MITRG AD mouse model [399].

One of the biggest advantages of iPSCs is the possibility to genetically modify the DNA sequence using gene-editing technologies, with clustered regularly interspaced short palindromic repeats (CRISPR)/Cas9 being the most widely used. Researchers can obtain cells from healthy or diseased donors to study aspects of genes involved in a particular disease. Importantly, another advantage of iPSC technology is the use of isogenic control lines, which do not carry the gene mutation of interest, but are otherwise identical to the iPSC cells with the disease genotype. One example of the power of this technology is a study that utilized CRISPR/Cas9 to modify APOE3 alleles in iPSCs from a healthy individual to APOE4, with APOE4 allele being the most significant AD risk gene [400]. Notably, APOE4 iPSC-derived microglia exhibited altered morphologies, which correlated with reduced phagocytosis of Aβ oligomers [401]. On the other hand, reporter lines to specifically label selected cell populations can also be applied to iPSCs, for instance, utilizing NF-κB tagged with green fluorescent protein reporter gene to recognize and monitor activated microglia in live models [402].

Another issue to keep in mind with iPSC cultures is that most neurodegenerative diseases develop at adult or elderly age and, therefore, it would be desirable to recapitulate age-related characteristics in microglia at the same age. However, reprogrammed iPSCs from adult donors have had their aging signature, such as telomere attrition and cellular senescence, reset. Direct reprogramming of somatic cells to microglia might address this problem by avoiding reprogramming to the stem cell state. In this sense, it has been demonstrated that direct reprogramming retains aging-associated transcriptomic signatures [403,404].

As we have discussed, there are a multitude of protocols allowing the evaluation of iPSC-derived microglia functionality in different settings, but, depending on the context, each one of them may be more or less suitable to the studied human condition. Over the next few years, we can expect an improved development of these protocols for the production of human microglial cells. It is clear to us that human iPSC-derived microglia represent an important tool in medicine for understanding the functional consequences of an increasing number of disease-associated risk factors linked to these cells [405]. They will likely also contribute to an improved therapeutic outcome for CNS diseases [406].

## 8. Conclusions

Since the microglia field of study commenced over a century ago, many exciting features have been discovered showing how truly multitalented microglia cells are [14]. In this review, we have tried to summarize different aspects of pro-inflammatory microglia, but many gaps still remain in our understanding of microglia biology. The idea that inflammatory responses driven by microglia are similar regardless of disease or stimulus has been shown not to be accurate. A recent study comparing genome-wide transcriptional responses of myeloid cells in the CNS under various conditions including aging, various neurodegenerative diseases, and inflammatory stimuli induced by LPS showed the existence of independent gene modules that could be grouped as either interferon-related, proliferation-related, or what the authors called “core neurodegeneration-related” [24]. This could be one of the reasons why, despite the established importance of microglia in the chronic neuroinflammatory response in AD, clinical trial results using nonsteroidal anti-inflammatory drugs (NSAIDs) have not been as successful as expected [407,408,409]. Another reason may be that we are focusing on only one aspect of the inflammatory response (in this case COX2 inhibition [407]), which might not be enough to achieve a neuroprotective outcome. In this sense, it may be more beneficial to modulate aspects of pro-inflammatory microglia such as we have discussed in this review. Various HDACi have anti-inflammatory and neuroprotective effects in vivo and are used frequently as cancer treatments because of the specificity of HDACi for cancer cells. However, as a treatment for chronic diseases, such as AD, there are concerns over the possible side effects of HDACi with prolonged treatment. Metabolic reprogramming of microglia during disease could also provide a novel treatment avenue to reduce microglia activation [301,410]. Some recent studies demonstrate that treating animals with either CX3CL1 (fractalkine) or IFN-γ can affect microglia metabolic reprogramming, change their phenotype, and confer neuroprotection under ischemic and AD models, respectively [301,305], but this will require more research for translation to the clinic.

Recent new techniques not yet implemented in microglia, such as chemogenetics and optogenetics, hold great potential to manipulate brain cell functions with temporal and spatial precision [411,412] and could shed light on microglia function. Both methods pursue the reversible control of selected signaling pathways in genetically defined cell populations with the help of chemical ligands or light stimulation of light-sensitive proteins, respectively. Mostly performed in neuronal populations, they are also starting to be used in glial cell populations, having been found useful in the study of astrocytic functions [413]. In the future, with the development of effective targeting strategies to microglia, they can be an additional tool in the research arsenal leading to better understanding of microglia function and behavior that may lead to new translatable therapeutic targets for neurodegenerative diseases.

## Figures and Tables

**Table 1:** TLRs expressed in microglia (human (H) and mouse (M)) and ligands/agonists classified in PAMPs and DAMPs.

TLR	Expression Level		PAMPs	DAMPs
	M	H		
1	+	+++	Lipoproteins [67]	α-syn [68]
2	+++++	++++	PG [67,69], lipoproteins, LTA, zymosan [67], synthetic bacterial lipopeptide Pam3CysSK4 [70]	α-syn [68,71], Aβ [72], gangliosides, hyaluronic acid [73]
3	+	++	Poly(I:C), viral dsRNA [67,74]	mRNA from apoptotic cells [75], stathmin[76]
4	+	++	LPS [77], monophosphoryl lipid A [78]	Aβ [79], α-syn [80,81], MPP^+^ [82], HSP60, fibrinogen [73]
5	+	+	Bacterial flagellin [67]	ND
6		+	PG [69], lipoproteins, LTA [67]	HMGB1 [83]
7	++++	++	Loxoribine [84], miR [85], ssRNA [67,86]	Self RNA, microRNA [83]
8	+	+	ssRNA [67]	Self RNA, microRNA [83]
9	++++	+	CpG-DNA [87,88], CpG-ODN [84,89], bacterial DNA [67]	DNA degenerating neurons [90], HMGB1 [91]
10	NF	+	ND	ND
11	+	NF	Profilin [92]	ND
12	+	NF	ND	ND
13	++	NF	Bacterial RNA [93]	ND

Abbreviations: CpG-DNA, cytosine-guanosine dinucleotides; CpG-ODN, cytosine-guanosine oligodeoxynucleotide; dsRNA, double-stranded RNA; HSP60, heat shock protein 60; LTA, lipoteichoic acid; MPP^+^, 1-methyl-4-phenylpyridinium; NF, not found; PG, peptidoglycan; Poly(I:C), polyinosinic-polycytidylic acid; ssRNA, single-stranded RNA. Legends: + (0.1-10.0 (fragments per kilobase of transcript per million mapped reads) FPKM), ++ (10.1-30.0 FPKM), +++ (30.1-60.0 FPKM), ++++ (60.1-90.0 FPKM), +++++ (>90.0 FPKM). Data source: https://www.brainrnaseq.org/. RNAseq data for mouse and human microglia obtained from [94,95], respectively.
